# Functionalized Liposome and Albumin-Based Systems as Carriers for Poorly Water-Soluble Anticancer Drugs: An Updated Review

**DOI:** 10.3390/biomedicines10020486

**Published:** 2022-02-18

**Authors:** Sofia Teixeira, Maria Alice Carvalho, Elisabete M. S. Castanheira

**Affiliations:** 1 Centre of Chemistry, Campus de Gualtar, University of Minho (CQUM), 4710-057 Braga, Portugal; id9191@alunos.uminho.pt (S.T.); mac@quimica.uminho.pt (M.A.C.); 2 Centre of Physics of Minho and Porto Universities (CF-UM-UP), Campus de Gualtar, University of Minho, 4710-057 Braga, Portugal

**Keywords:** nanocarriers, functionalized liposomes, albumin-based nanosystems, poorly water-soluble drugs, targeted cancer therapy

## Abstract

Cancer is one of the leading causes of death worldwide. In the available treatments, chemotherapy is one of the most used, but has several associated problems, namely the high toxicity to normal cells and the resistance acquired by cancer cells to the therapeutic agents. The scientific community has been battling against this disease, developing new strategies and new potential chemotherapeutic agents. However, new drugs often exhibit poor solubility in water, which led researchers to develop functionalized nanosystems to carry and, specifically deliver, the drugs to cancer cells, targeting overexpressed receptors, proteins, and organelles. Thus, this review is focused on the recent developments of functionalized nanosystems used to carry poorly water-soluble drugs, with special emphasis on liposomes and albumin-based nanosystems, two major classes of organic nanocarriers with formulations already approved by the U.S. Food and Drug Administration (FDA) for cancer therapeutics.

## 1. Introduction

Cancer is one of the leading causes of death worldwide. In 2020, 18.9 million new cases occurred, and cancer caused 10.1 million deaths worldwide. By 2040, the expected number of new cases is 29.5 million, and the number of cancer deaths, 16.4 million [1,2].

Cancer consists of uncontrolled growth, the invasive spread of abnormal cells, and genome mutations [3,4,5]. Moreover, abnormal cells can disseminate to other parts of the body, which is called metastasis [3]. Surgery, radiation, and chemotherapy are conventional cancer treatment methods. Surgery implies the removal of the tumor mass and is widely used. Chemotherapy is also one of the foremost and most common therapeutic methods in cancer, also being employed in combination with surgery or radiation [5,6]. Chemotherapy uses chemical agents to target rapidly growing and dividing cells, due to the fact that cancer cells grow faster than healthy ones, treating the entire body [3,7,8]. However, due to the lack of specificity of conventional chemotherapeutic agents, they act in both types of cells, destroying many non-cancerous cells, leading to severe side effects and high toxicity, which, in turn, leads to a low quality of life for patients [5,8].

Although conventional therapies are necessary, they do not always perform well for all patients, besides the strong side effects, and so new therapeutic approaches have been developed to target cancer. Nevertheless, chemotherapy is the most suitable option to treat cancer, alone or in combination with other therapies [3,4]. Many of the drugs (already used in chemotherapy or new ones) have poor water solubility and, therefore, cannot be delivered efficiently to cancer cells [9,10].

Cytotoxic agents’ resistance is another serious problem in cancer therapy. Cancer cells acquire drug resistance by modifications in drug metabolism and transport, gene mutation, amplification of drug targets, and genetic rewiring, leading to gene repair and impaired apoptosis [4,5]. Thus, it is common in traditional chemotherapy that drugs do not specifically target and kill cancer cells, leading to the development of resistance to treatment, ultimately producing a more aggressive cancer. Despite several advances in drug discovery and treatment protocols, patients acquire frequently multidrug resistance (MDR). This MDR occurs when tumor cells develop resistance to functionally and structurally unrelated anticancer drugs. This happens because cancer cells initially respond to chemotherapy but relapse; in fact, tumor cells manage to escape the effects of the cytotoxic drugs during or shortly after the therapy. Therefore, MDR is a major obstacle to successful chemotherapy [5,6,7]. 

The ultimate goals of cancer therapy are to overcome resistance, to reduce systemic toxicity and improve the patients’ quality of life. The need for site-specific delivery systems with low systemic toxicity is the current challenge of anticancer drug development. Thus, the limitations of chemotherapy led to the development of nanocarrier-based drug delivery systems (DDS) [5,6,11]. 

Nanocarriers comprise several architectures to transport substances. Conventional nanocarriers cannot carry and release drugs with the right concentration at the targeted site, under external or internal stimulation. The solution is the development of smart nanocarriers, also known as Smart Drug Delivery Systems (SDDS), by modifying and functionalizing conventional drug carriers, aiming to allocate drugs to specific and targeted sites [5,7].

In this review, the different types of nanocarriers for chemotherapeutic agents are summarized, with a major emphasis on liposomes and on albumin-based nanocarriers, two major classes of DDS, with approved formulations by the U.S. FDA. This manuscript is focused on the ability of these nanosystems to enhance the solubility of new drugs and to promote targeted therapies through surface functionalization, describing recent developments and successful examples of these approaches. This review constitutes an updated (last 5 years), detailed and comprehensive review on functionalized nanosystems, developed to transport and deliver poorly water-soluble drugs in cancer therapy. It can be a guide for medicinal chemists to help in the selection of an appropriated nanocarrier for new drugs in development that present issues of water solubility. 

## 2. Nanocarriers for Delivery of Poorly Water-Soluble Drugs in Cancer Therapy

Poor water solubility is a common problem in the new drug candidates, caused by a high lipophilicity, large molecular weight, and high logP values [12,13]. About 40% of new drugs with market approval and 60% of new chemical entities coming directly from chemical synthesis are poorly water-soluble [13,14]. Most of the drugs are either weakly acidic or weakly basic, which also contributes to poor aqueous solubility. Despite their potential pharmacological activity, new drugs with poor water solubility cannot be successfully launched in the market [15].

New, poorly soluble drug candidates are limited in their real application, because low solubility reduces the dissolution rate and oral bioavailability, creating a challenge in pharmaceutical development [14,15,16,17]. Moreover, many new drugs fail to meet appropriate absorption, distribution, metabolism, and excretion (ADME) properties, due to the low dissolution rate, resulting in low blood levels [12]. In order to obtain an ideal molecule, besides the pharmacological activity, it is necessary to have appropriate physicochemical characteristics, such as low toxicity, associated with selectivity to the therapeutic target, slow metabolism, and solubility in water [15]. Consequently, research is focused on the development of more effective and versatile approaches, comprising chemical and physical modifications, to improve the bioavailability of hydrophobic drugs [12]. To enhance bioavailability and absorption, an increase in the dissolution rate is required, which is possible with nanonization, by increasing the particles’ surface area and saturation solubility. This approach uses a low amount of excipient, thereby reducing the potential toxicity [13,14,15,16,18]. 

The advancement of nanomedicine (the medical application of nanotechnology) allowed researchers to develop nanocarriers, such as nanoparticles or nanostructures (carrying materials or not) that incorporate drug molecules [19,20,21]. Therefore, modern medicine can make use of nanosystems to deliver bioactive compounds with a sufficient dose of a drug [19,20,21,22,23,24,25,26].

**Figure 1:**
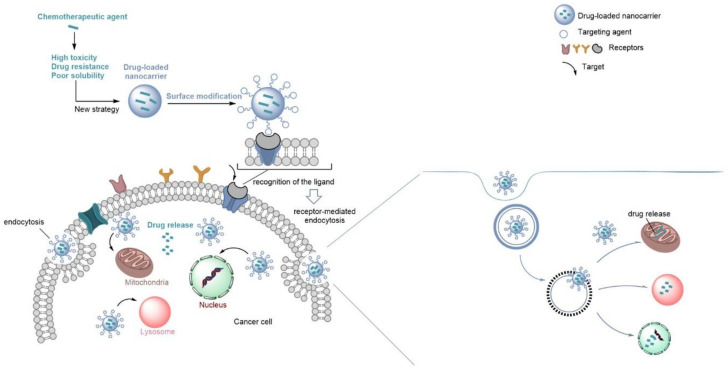
Representation of the active targeting of cancer cells by functionalized nanocarriers. Specific ligands on the nanocarrier surface bind to cell surface receptors. Upon recognition of the ligands, the uptake of the functionalized nanocarriers occurs by receptor-mediated endocytosis. The nanocarriers can also be functionalized with ligands to target specific cell organelles.

Nanocarriers are generally classified in two major groups, organic and inorganic nanoparticles [13,27,28]. Organic nanocarriers include lipid-based nanoparticles (NPs) and polymeric nanocarriers, while inorganic nanocarriers comprise carbon nanotubes (CNTs) and mesoporous silica nanoparticles (MSNs) [28,29]. Some of the nanocarriers are recognized for their simplicity, biocompatibility, and non-toxicity, but also for the capacity to enhance the solubility, absorption and bioavailability of the drugs. In addition, researchers use these nanocarriers to achieve the best therapeutic effect by employing the smallest dose of drugs, in suitable dosage and mode of administration. However, nanocarriers do not always achieve their purpose, since their efficacy depends on size, size distribution and shape. They could also accumulate in some organs, such as the liver, kidney, spleen, and lungs, not reaching the required concentration of compound at the intended location. Moreover, sometimes the drug encapsulation efficiency is low, which implies the use of a larger amount of compound. In some cases, the nanocarriers show burst or disrupted release [30,31,32,33,34,35,36,37,38,39,40,41,42]. Nanomedicines can own properties that make them an attractive option in therapy. In fact, nanocarriers expand the application ranges of hydrophobic drugs, deliver drugs at disease sites through passive targeting, such as the enhanced permeability and retention (EPR) effect [43], or through active targeting with specific nanoparticle–cell surface interaction, due to coatings and functionalization of nanocarriers by molecules [44,45] (Figure 1). These specific nanostructures promote longer circulation times of drugs when dosed into the circulatory system, allow controllable release [46,47] and lower systemic toxicity [48,49], as well as a higher drug bioavailability [50]. Moreover, it is possible to have real-time monitoring of the carrier (and drug) biodistribution and targeted accumulation [20,21,25]. To overcome poor water solubility and improve oral bioavailability [15,16,51,52], effective oral drug delivery systems, such as lipid-based drug delivery systems [31,53,54], polymeric micelles [55], and inorganic nanoparticles [16,56], have been used. Due to their success in carrying hydrophobic drugs, this review in mainly focused on lipid and albumin-based nanocarriers. The reported studies with functionalized nanocarriers solved some of the problems associated with the conventional nanocarriers, but in some, further studies are needed.

### 2.1. Lipid-Based Nanoparticles

The most common class of nanomedicines approved by the U.S. FDA is the one of lipid-based nanoparticles. Lipid-based nanoparticles comprehend multiple structures, most of them being typically spherical, with at least one lipid layer and one aqueous core [29,57]. Lipid nanoparticles possess, as advantages, simplicity, self-assembly, high biocompatibility and bioavailability, great ability to carry large quantities of cargo, and a variety of physicochemical properties that can be controlled to modulate their biological characteristics [29,58].

#### 2.1.1. Liposomes

Liposomes are drug-delivery vehicles, introduced in the 1970s, with a size range between 10 nm and 1 μm. They consist of vesicular systems and thermodynamically stable spheres, with an aqueous core enclosed by one or more phospholipid bilayers. These nanosystems have been widely used to encage, carry and deliver hydrophilic, hydrophobic and amphiphilic drugs, separately or together [15,20,26]. Hydrophilic and hydrophobic drugs should allocate in the aqueous compartments and lipid bilayer, respectively [15]. 

Liposomes are excellent therapeutic carriers [59,60] due to several advantages, such as the following: they are biodegradable and biocompatible [61]; they can enhance and sustain the delivery of drugs [43,62], enhancing therapeutic efficacy; improve drug pharmacokinetic properties, in comparison to free drugs in solution [51]; protect the active drug from abiotic factors, preventing degradation [63]; decrease systemic toxicity of the encapsulated drug [64]. By contrast, they may lose bioactive compounds and have short release times; they may be opsonized by plasma proteins (opsonins) and uptaken by the mononuclear phagocyte system. Furthermore, the production costs of liposomal formulations have been generally high [26]. In a recent study, Bhatt et al. [35] produced two PEGylated liposomal formulations and compared with Taxol^®^, to improve loading, delivery, and prolong the release of Paclitaxel (PTX), since the premature and rapid release, in vivo, of hydrophobic compounds appears to be a problem in conventional liposomes. In fact, the authors demonstrated that PTX release of Taxol^®^ was about 92% within 24 h, at pH = 7.4, which was a rapid release when compared with the two other formulations, with a PTX release of about 39 and 28%. This study provides evidence that some liposomal formulations can have premature release. However, the new produced liposomes could indeed prolong the release profile, circulation time, and plasma concentration, which allowed obtaining higher cytotoxicity in cancer cell lines. Moreover, even with modification of the liposomes, the drug in some cases is partially released into the blood circulation after intravenous injection, not achieving the total concentration in the desired site [65].

**Figure 2:**
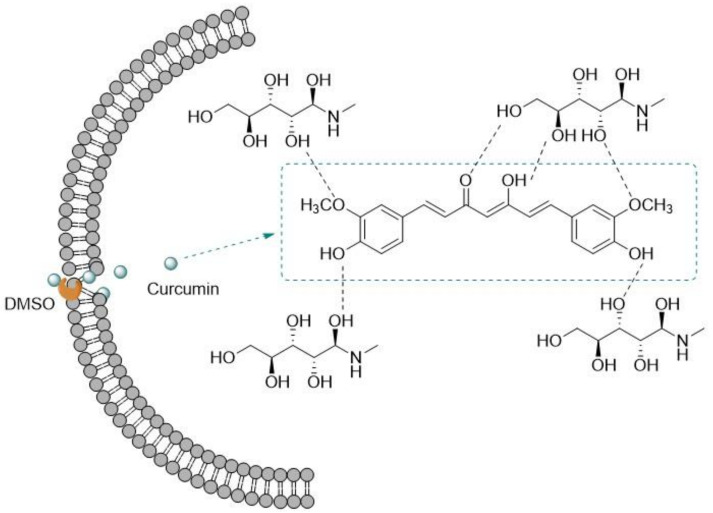
Schematic representation of SALT method for liposome preparation. DMSO is used as solvent for curcumin, to increase bilayer permeability and allow active curcumin loading into the inner aqueous core of liposomes. Formation of hydrogen bonds between the buffers used (Meglumine or Myo-inositol) and curcumin allows trapping the drug in the aqueous core. Adapted from [36] with permission from Elsevier, 2022.

Liposomes have been extensively used to carry drugs with poor water solubility with great results [36,66,67,68,69,70,71]. For example, Karimi et al. [36], with the aim of enhancing drug loading capacity with high stability, improved bioavailability, and enhanced tumor accumulation of the poorly water-soluble drug curcumin (CUR), developed stable liposomal formulations of CUR by the solvent-assisted active loading technology (SALT) method, which has the advantage of active and efficient loading of CUR in the core of liposome (Figure 2). The study showed that liposomal formulations carrying the drug increased its half-life, in comparison with free CUR. This could be due to a prolonged circulation of PEGylated liposomes (containing polyethylene glycol, PEG, at surface), and the increased chance of tumor accumulation by the EPR effect, promoted by the incorporation of negatively charged phospholipids, such as DPPG. These liposomes exhibited an improved physical stability and drug loading capacity. However, the authors verified that CUR release from the nanoliposomes was low (21–22%) [36].

#### 2.1.2. Solid Lipid Nanoparticles

In the 1990s, a group of researchers developed an alternative lipid-based carrier, the solid lipid nanoparticles (SLNs) [72,73,74]. The SLNs have sizes between 50–1000 nm and were formulated by replacing the liquid lipid of emulsions with a solid one at room temperature, as well as at body temperature [26,75]. These SLNs are composed of physiologically tolerated lipids, dispersed in an aqueous surfactant phase, including a highly lipophilic matrix, prepared with solid lipids, such as mono-, di- and triglycerides, fatty acids, and steroids. The choice of lipids should be based on drug solubility in the lipid material. These nanocarriers can incorporate the active substance in the particle core, in the shell or it can be dispersed within the whole lipid matrix [75,76]. The solid lipid core makes it possible to solubilize lipophilic compounds and the surfactants/emulsifiers stabilize it [26].

The SLNs allow enhanced pharmacokinetic properties and modulate the release of drugs [77,78], protecting them from chemical degradation; they increase permeation through the biological barrier [79,80]; they have the possibility of surface modification [81] and co-delivery of several therapeutic agents; allow controlled drug delivery; exhibit absence of biotoxicity; promote the bioavailability of poorly water-soluble drugs [53,82,83]. Besides, this carrier system can increase the specificity and decrease the cytotoxicity of conventional anticancer therapy, proving that SLNs improve the therapeutic efficacy of anticancer drugs [84,85]. However, SLNs have some limitations, such as reduced loading capacity, stability problems and agglomeration during storage, high operative temperature and low circulation time [75]. 

Recently, SLNs have been used for cancer therapy to improve solubility and make use of targeted therapy [53,83,86]. For example, Rodenak-Kladniew et al. [86] encapsulated Linalool (LN), a compound with poor water solubility, in SLNs with different compositions and tested their activity in vitro, in two cancer cell lines (HepG2 and A549 cells). They verified that SLNs of cetyl esters with the encapsulated drug improved the inhibitory efficiency in HepG2 cells and SLNs of myristyl myristate with LN showed in vitro antiproliferative effects, on both cell lines in a dose-dependent response, with higher inhibitory effects when compared to free LN.

**Figure 3:**
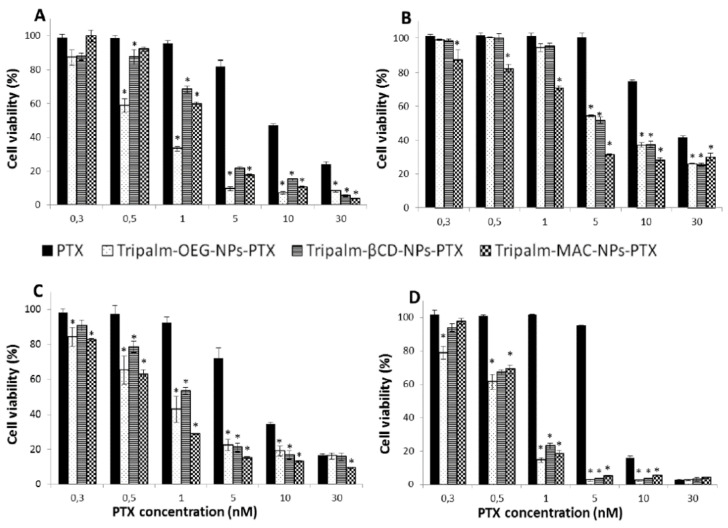
Cytotoxicity of modified Tripalm-NPs-PTX with MAC, OEG and β-CD. Cell viability (%) of breast cancer MCF7 cells (**A**), corresponding normal cells MCF-10A (**B**), lung cancer A549 cells (**C**) and non-tumor cells L132 (**D**), after treatment with modified Tripalm-NPs-PTX and free PTX. Data represent the mean value ± SD of quadruplicate cultures. (*) Significant differences (*p* < 0.001) between free PTX and modified Tripalm-NPs-PTX. Reprinted from [85] with permission.

Furthermore, Leiva et al. [85] developed solid lipid nanoparticles of glyceril tripalmitate, loaded with Paclitaxel (PTX) (Tripalm-NPs-PTX), including modifications by the addition of hexa(ethylene glycol) (OEG), β-cyclodextrin (β-CD) and macelignan (MAC). All the formulations significantly enhanced PTX antitumor activity against human breast and lung cancer cells (Figure 3).

Smith et al. [77] developed optimized SLNs, capable of delivering a high payload of 5-Fluorouracil (5-FU), consisting of PEGylated SLNs with precirol as lipid phase and a mixture of Tween^®^-80/Lecithin (4:2) as the surfactant composition. This drug delivery system enhanced the entrapment efficiency and improved 5-FU pharmacokinetic parameters, with a high cytotoxicity against HCT-116 cells, significantly inhibiting subcutaneous tumor growth in mice, when compared to free 5-FU. 

#### 2.1.3. Micelles

Micelles are colloidal suspensions, formed by amphiphilic compounds with small diameters, ranging from 5 to 100 nm. Micelles can carry hydrophobic and hydrophilic drugs in nanosized structures, such as spherical, ellipsoid, cylindrical, or unilamellar. Micelles might have very low critical micelle concentration (cmc) when formulated with lipids. Hence, alternative amphiphilic materials, such as amphiphilic copolymers, have been developed [87]. 

Micelles have some advantages to deliver anticancer drugs. They can prolong blood circulation [88]; can save the water-insoluble drugs in their hydrophobic core because hydrophobic regions of amphiphilic molecules create cavities or nucleus [89]; sometimes, they can improve the water solubility of bioactive agents [89,90]. Also, these nanocarriers enhance the bioavailability and therapeutic efficacy of encapsulated agents and reduce toxicity [87,91]. Nevertheless, micelles also have disadvantages, such as reduced stability in the bloodstream, where critical micelle concentration could be reduced by blood dilution and minimized drug circulation half-life [87].

**Figure 4:**
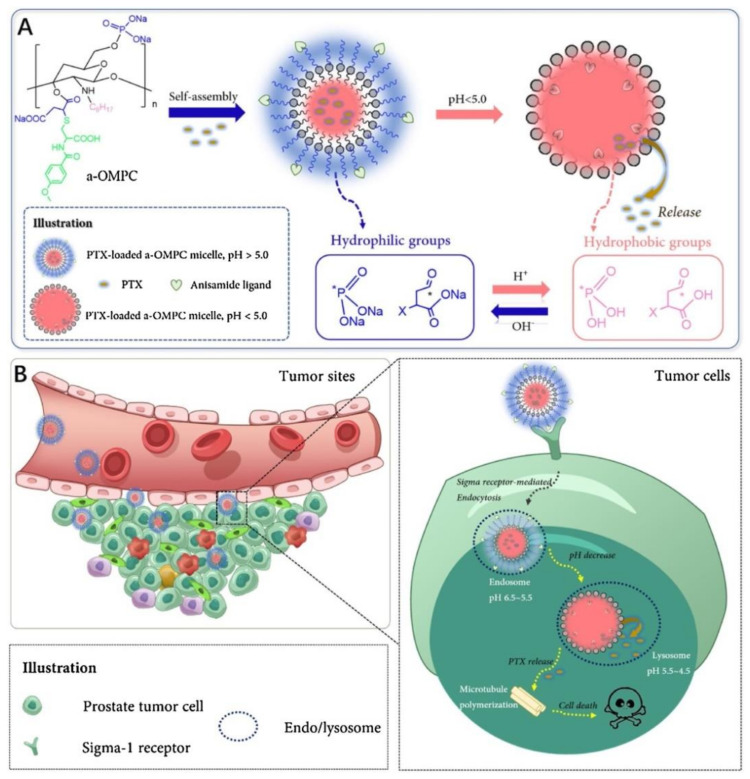
Representation of the synthesis of PTX-loaded a-OMPC micelles (**A**); Cellular internalization with recognition of the PTX-loaded a-OMPC micelles by sigma-1 receptor overexpressed in prostate tumor cell, and the release of the PTX by pH-responsive release (**B**). Reprinted from [37] with permission from Elsevier, 2021.

Recently, micelles have been used to improve the transport of therapeutic agents like PTX. Qu and co-workers [37] synthesized an anisamide-conjugated *N*-octyl-*N*,*O*-maleoyl-*O*-phosphoryl chitosan (a-OMPC), which can form amphiphilic micelles for PTX delivery (Figure 4A). In this study, they demonstrated that PTX-loaded a-OMPC micelles enhanced the cellular internalization and increased the cytotoxicity against PC-3 cells, due to the high affinity to sigma-1 receptor overexpressed tumors and pH-responsive release, respectively (Figure 4B). Despite the possible accumulation of the compound at the liver and spleen, even after the treatment with micelles, the undesired accumulation was diminished. Also, these micelles could largely accumulate at the tumor site.

Chen et al. [90] developed amphiphilic conjugates, associating curcumin to a food-derived hydrophilic hydroxyethyl starch (HES), via an acid-labile ester linker. The nanomicelles showed desirable drug loading efficiency, excellent colloidal and storage stability, as well as an acid-responsive release manner. The nanoparticles increased the solubility of curcumin, in comparison to free drug, and effectively protected the loaded drug from degradation. Also, the authors verified enhanced solubility and bioavailability, which can be the cause of the significantly improved cytocompatibility, anticancer and antioxidant activity of the drug achieved with this nanocarrier. 

### 2.2. Polymeric Nanoparticles

**Figure 5:**
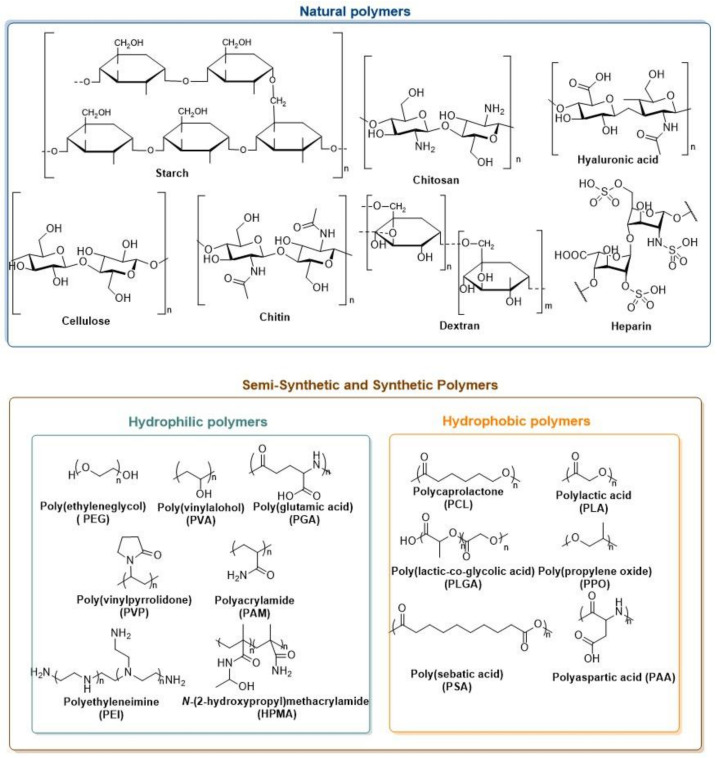
Structural representation of some natural, semi-synthetic and synthetic polymers.

Polymeric nanoparticles are solid, nanosized (10–100 nm) colloidal particles [92,93,94]. These nanoparticles can be obtained from natural polymers (Figure 5), such as albumin, starch, cellulose, chitin, chitosan, hyaluronic acid, collagen, dextran, gelatin, laminin and heparin, or even from semisynthetic or synthetic polymers (Figure 5), including hydrophobic ones (polylactic acid (PLA), polycaprolactone (PCL), poly(lactic-co-glycolic acid) (PLGA), poly(propylene oxide) (PPO), polyaspartic acid (PAA)) and hydrophilic polymers (polyglycolic acid (PGA), PEG, *N*-(2-hydroxypropyl)methacrylamide (HPMA) copolymer, and polyglutamic acid). These nanoparticles can be produced by a polymerization reaction and self-assembly [94,95].

These nanoparticles can carry anticancer drugs (both hydrophobic [96] and hydrophilic [97]), either entrapped, encapsulated or bound, in a wide variety of possible structures, such as nanospheres, nanocapsules or drug conjugates. Nanospheres entrap the drug inside their matrix by physical dispersion or adsorption on the particle surface, while nanocapsules have a polymeric shell, surrounding a core cavity that encapsulates the drug [95]. Polymeric capsules can possess targeting ligands to increase selectivity for cancer cells and improve intracellular drug delivery, along with the decrease in side effects and drug toxicity [98,99,100,101,102,103]. 

Natural polymers are highly stable and safe, comprising different classes of polysaccharides and proteins. Despite the possibility of synthetic polymers’ structures being designed and their physicochemical properties being modified, natural polymers can be functionalized with synthetic molecules [104]. On the other hand, synthetic polymers used in drug delivery systems should have stability in blood circulation, low toxicity, be activated when needed, and should protect the drugs against early degradation in the target tissue [95]. Natural and synthetic polymers are usually biocompatible and biodegradable [92,93], polymeric nanoparticles being promising delivery vehicles for therapeutic agents [95]. Furthermore, they disintegrate into individual monomers inside the body and, hence, are removed from the body through normal metabolic pathways [37].

Polymers for the delivery of therapeutic agents were introduced in 1975 [105] and polymeric nanoparticles (NPs) are used nowadays in cancer therapy, involving different architectures, such as polymeric micelles, nanocapsules, dendrimers, polymersomes, polymeric nanogels and polymer–drug conjugates [97,103,106,107,108,109,110]. Polymeric NPs are advantageous as carriers for chemotherapeutic drugs, as they are water-soluble [29]; can improve the drug bioavailability and therapeutic efficacy; can increase drug circulation time [96,103,111,112]; allow controlled release and protect loaded drugs from degradation [93,113]; are generally stable [97]; allow a high drug payload and controllable physicochemical properties [28].

The earliest attempt to deliver a hydrophobic anticancer drug, DOXorubicin (DOX), using polymer nanosystems was made in 1979 [28]. Recently, polymer–drug conjugates have been designed to improve the water solubility of the conjugated drug. Luo and co-workers [114] developed PEG–PTX conjugates, with enhanced solubility of PTX. Chen et al. [41] also reported an improved solubility of this drug when conjugated with C-6 hexanediamine-modified hyaluronic acid (HA-6). The authors verified that PTX release was 20% after 96 h, at pH 7.4, and was even lower at pH 6.0, with a PTX release of 12%. However, when the conjugate was placed at pH 6.0 with hyaluronidase, the release behavior shifted to around 42% after 96 h. Despite that, in this study, the nanocarrier showed increased cytotoxicity and an enhanced apoptosis-inducing effect against HepG2 and A549 cells, due to the increased cellular uptake of the drug. 

Albumin-based nanoparticles have been reported as an important DDS for poorly water-soluble drugs, given that albumin holds high binding capacities for hydrophobic substances [33,46,115,116,117,118,119]. For example, Saleh et al. [118] increased the solubility of curcumin by developing CUR-loaded human serum albumin (HSA) nanoparticles and obtained an enhanced anti-tumor effect, with a targeted aptamer-decorated CUR-loaded HSA nanoparticle. 

### 2.3. Inorganic Nanoparticles

Inorganic nanoparticles are composed of several types of inorganic materials, such as iron oxide, gold, zinc, silver, and silica nanoparticles, or even carbon nanotubes and graphene [120,121]. These nanoparticles can possess magnetic, catalytic, electrical and specific optical properties [120]. Inorganic nanoparticles can serve as drug delivery vehicles due to their capability to extend the retention of drugs in blood circulation, to enhance drug accumulation in tumors and therapeutic efficacy, to increase the bioavailability and uptake by cancer cells and avoid unfavorable side effects. Also, these NPs exhibit a high surface-to-volume ratio, controllable size and shape, and can suffer surface modifications [121]. However, the potential toxicity of inorganic nanoparticles is the major obstacle during the clinical translation [122].

**Figure 6:**
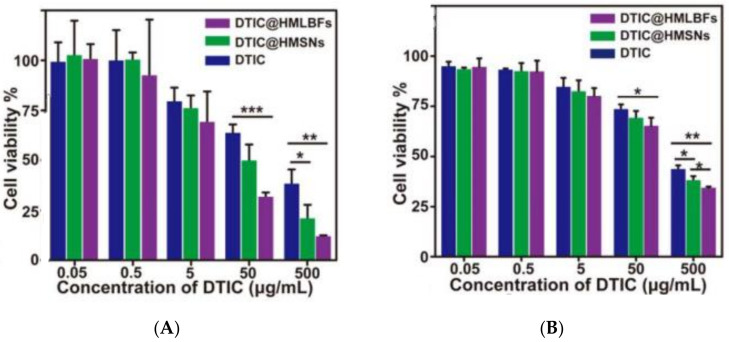
Cell viability of (**A**) A875 and (**B**) B16/F10 cells after incubation with different concentrations of DTIC@HMLBFs, DTIC@HMSNs, and free DTIC for 48 h. The experiments were conducted in triplicate (* *p* < 0.05, ** *p* < 0.01, *** *p* < 0.001, results were given as Mean ± SD). Reprinted from [56] with permission from American Chemical Society, 2021.

**Figure 7:**
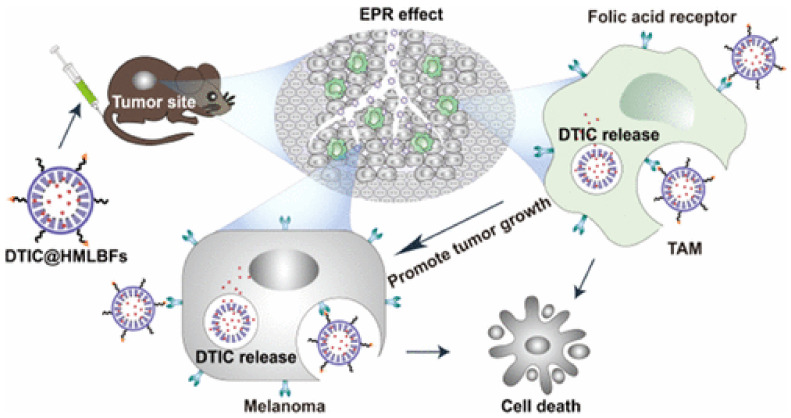
Schematic representation of in vivo targeting with nanocarrier DTIC@HMLBFs. First, by EPR effect, the nanocarrier can achieve the tumor tissue, with higher local accumulation, and then target folate-receptors of melanoma cells and tumor-associated macrophage (TAM). Reprinted from [56] with permission from American Chemical Society, 2021.

Liu et al. [56] reported a promising nanocarrier for delivering the anticancer drug Dacarbazine (DTIC). This drug is an important chemotherapeutic agent for the treatment of melanoma, but has poor solubility, photosensitivity, stability and serious toxicity to normal cells. Hence, these authors developed hollow mesoporous silica nanoparticles (HMSNs) for the encapsulation and release of DTIC, by modifying MSNs with carboxyl groups to enhance drug loading, followed by the further coating with folic acid-grafted liposomes (DTIC@HMLBFs) for controlled and targeted drug release. The studies in vitro revealed that DTIC@HMLBFs exhibited the strongest cytotoxicity to melanoma cells, compared to non-targeted nanoparticles and free DTIC (Figure 6). The in vivo studies showed that the nanocarrier, loaded with DTIC, achieves significant improvement against lung metastasis of melanoma, via passive and active targeting of melanoma cells and tumor-associated macrophage (TAM) (Figure 7). 

## 3. Nanocarriers for Targeted Cancer Therapy

In order to overcome the multiple serious problems of conventional chemotherapy, new anticancer agents based on targeting strategies have emerged. These strategies involve nanotechnology and bioconjugation chemistry, which can alter drug biodistribution to avoid toxicity, maximize its efficacy and target selectivity [123,124,125]. The drug release in the tumor can occur by a DDS, targeted to specific organs, where the tumor is residing, or even specifically, to the cancer cell surface [126,127]. Delivering chemotherapeutic drugs directly into cancer cells is crucial, avoiding premature drug release and decreasing systemic toxicity [5,125]. Moreover, the cellular efflux mechanisms cannot recognize anticancer agents encapsulated in nanoparticles; hence, it is more difficult for multidrug resistance to occur [124].

Targeted drug delivery uses specific target molecules and drug delivery systems conjugated to ligands [127,128]. Liposomes [31,129] and albumin-based systems [130,131] are examples of the most used nanocarriers in targeted therapy, improving the intracellular delivery of chemotherapeutic agents by active targeting. Passive targeting exploits characteristic features of tumor biology, such as systemic and lymphatic systems, allowing a selective accumulation of drugs at the tumor site through the EPR effect [7,132]. Active targeting combines nanocarriers containing the drug with molecules that bind specifically to overexpressed antigens or receptors on the target cells, meaning a ligand-mediated drug delivery. The ligands can be covalently conjugated to an active agent or be located on the surface of a carrier system. The targeting agents can be broadly classified as proteins (mainly antibodies and their fragments), nucleic acids (aptamers), or other receptor ligands (peptides, vitamins, and carbohydrates). Also, ion channels, such as potassium (K^+^), sodium (Na^+^), calcium (Ca^2+^), chloride (Cl^−^), and AQP4 channels, may be targeted to regulate tumor metastases [5,7,132].

Releasing drugs at the specific location and at a precise concentration is the subsequent step. Moreover, local drug accumulation can increase by carrying the drug within a nanosystem and control releasing it, when bound to the targets [7,132]. Many efforts have been made to increase the delivery and therapeutic efficacy of drugs by targeting specific receptors, proteins and organelles. This will be explained below, exploring the functionalization of liposomes (one of the most successful nanocarriers already introduced on the market [133]), but also describing functionalized albumin-based nanocarriers, since albumin can load and transport different drugs, including the hydrophobic ones [119].

### 3.1. Functionalized Liposomes for Cancer Therapy

**Figure 8:**
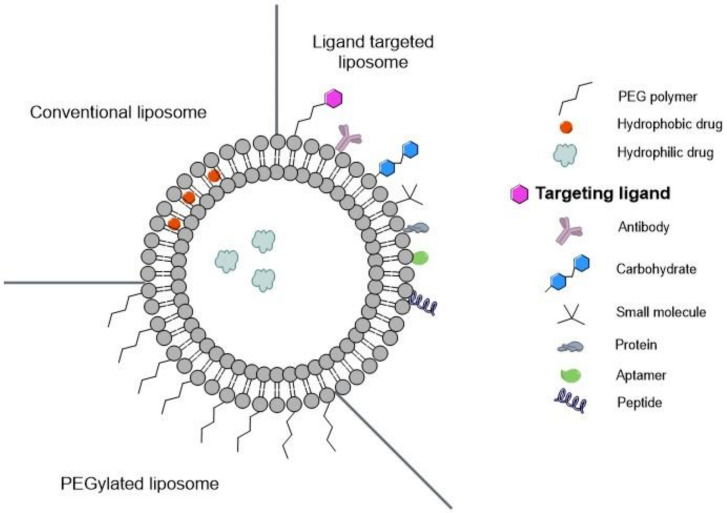
Different formulations of liposomes: conventional liposomes; PEGylated liposomes and ligand targeted liposomes for active targeting.

As referred to above, liposomes are pharmaceutical carriers for drugs (or genes) based on lipid bilayers surrounding an aqueous core. These nanosystems greatly facilitate the delivery of anticancer agents, because of their resemblances with the nature of the cell membrane [60]. Passive and active targeting strategies have been proposed to promote the delivery of drugs by liposomes and enhance the tumor-selective accumulation of anticancer agents [42,134,135]. In the past few years, the functionalization of liposomes’ surface has been used to target cancer cells (Figure 8), overcoming systemic toxicity and allowing longer circulation time and controlled release [129,134,136].

#### 3.1.1. Passive Targeting

Nanocarriers with size between 100 nm and 200 nm are passively accumulated in cancer cells via the EPR effect [135]. The accumulation of the liposomal nanosystems can be dramatically improved by extending their circulation time, which can be achieved by coating with polyethylene glycol. PEGylated liposomes exhibit unique properties of long circulating time, but can also be used with active targeting approaches [137]. The use of PEGylated DOX-loaded liposomes for passive targeting was approved by the U.S. FDA as Caelyx^®^. Even though Caelyx^®^ significantly improved the pharmacokinetics and half-life of DOX, the drug had low cellular uptake and a low release rate at the tumor sites [138]. Thus, Mashreghi et al. [135] proposed surface-functionalized PEGylated-nanoliposomal DOXorubicin, with an anti-EpCAM (epithelial cell adhesion molecule) aptamer to active targeting colon carcinoma C26 cells. The formulation showed an enhanced rate of cell uptake on C26 cells and enhanced cytotoxic effects compared with Caelyx^®^, but also improved the tumor accumulation of DOX. This example shows the limitations of the EPR effect and the need to consider active approaches or the combination of both.

#### 3.1.2. Active Targeting

As will be described below, active targeting approaches involve the preparation of targeted nanocarriers, by surface functionalization, with a suitable targeting ligand, as demonstrated previously in the case of liposomes (Figure 8).

- Targeting Overexpressed Receptors on Cancer Cells with Liposomes
  - Targeting Epidermal Growth Factor Receptor (EGFR)

The epidermal growth factor receptor (EGFR) belongs to human epidermal growth factor receptor 1, simultaneously with HER1 and ErbB1. Human epidermal growth factor receptors 2 includes HER2 and ErB2, both types of receptors belonging to the tyrosine kinase receptor family, which regulates cell proliferation and differentiation [139]. The EGFR is overexpressed in multiple cancers [140], remaining an important target in the delivery of chemotherapeutic agents. Liposomes can target the epidermal growth factor receptor by binding an antibody to the targeted site.

**Figure 9:**
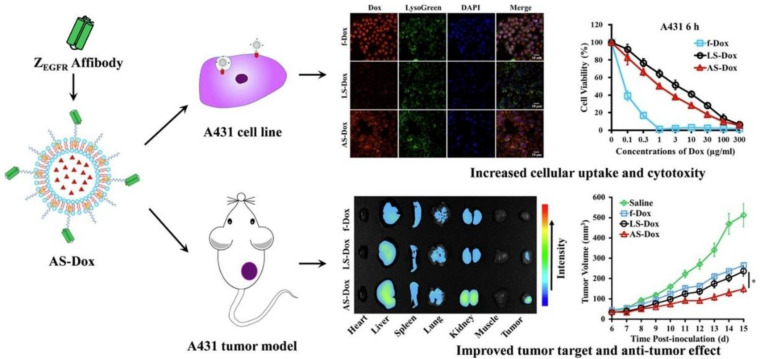
In vitro (above) and in vivo (below) application of PEGylated DOX-loaded liposomes coupled to a high-affinity EGFR-antagonist affibody (Z_EGFR_), named AS-DOX, to target A431 tumor cells overexpressing EGFR. LS-DOX: Non-targeted liposomes; f-DOX: free DOXorubicin; * *p* < 0.05. Reprinted from [134] with permission from Elsevier, 2021.

Jia et al. [134] developed a PEGylated liposome with DOX, coupled to a high-affinity EGFR-antagonist affibody (Z_EGFR_), named AS-DOX, to target A431 tumor cells overexpressing EGFR and compared to non-targeted liposomes loaded with the drug (Figure 9). The results showed that the nanocarrier, coupled with Z_EGFR,_ displayed a higher DOX uptake than non-targeted nanocarriers. Also, they verified that the IC_50_ value of targeted liposomes decreased approximately two-fold more than the non-targeted group against A431 cells, which proved that the cytotoxicity enhanced selectively in vitro. Moreover, the nanocarrier coupled with Z_EGFR_ had long circulation time and efficient accumulation in tumors in vivo.

To improve therapeutic efficacy, while minimizing the side effects of the anticancer agent celecoxib (CLX), Limasale et al. [42] developed EGFR-targeted immunoliposomes by using cetuximab, a monoclonal antibody, as a targeting ligand. The immunoliposomes only encapsulated 40% of the CLX, yet the amount of CLX was still enough to have anticancer activity. The uptake and cytotoxicity of immunoliposomes were higher than non-targeted liposomes in MDA-MB-468 cancer cells with EGFR-overexpression, with no toxicity against normal cells. Petrilli et al. [129] also adopted cetuximab as functionalization of liposomes, loaded with 5-FU, to selectively deliver this drug to squamous cell carcinoma (SCC) cells, verifying an increase in cellular uptake of cetuximab-immunoliposomes by SCC cells overexpressing EGFR.

- Targeting Transferrin Receptors

Transferrin receptors (TfR) are overexpressed in many cancers, such as brain, breast, colon, lung, pancreatic, and prostate cancers, due to an increased iron demand. Transferrin (Tf), a 76 kDa iron-binding glycoprotein, specifically binds to TfR on the surface of cancer cells [141]. The low amount of these receptors in most normal cells allows the development of functionalized nanocarriers targeting TfR.

Riaz et al. [142] studied a targeted nanocarrier to deliver Quercetin (QR), a drug with low water solubility and low bioavailability, to lung cancer cells. Quercetin-loaded liposome functionalization was reached by using T7 (HAIYPRH) peptide as the targeting ligand, due to its binding affinity for TfR. The results showed that both T7 surface-functionalized liposomes and non-targeted liposomes presented relatively higher cytotoxicity, as compared to free QR, probably due to the enhanced penetration ability of liposomes. A T7 peptide density of 2% in the formulation allowed significantly augmented cytotoxicity (~3-fold), with higher apoptosis induction and S-phase cell-cycle arrest, evidencing a successful strategy for targeted delivery.

Deshpande et al. [38] reported a promising nanocarrier to target A2780 ovarian carcinoma cells, via the overexpressed TfR, with an octaarginine cell penetrating peptide (R8), allowing intracellular DOX delivery. The DOX-loaded liposomes were surface-modified with two target ligands, R8 and Tf, making a dual targeted liposome (DOX-L). The nanocarrier showed better tumor penetration and intracellular delivery (by R8) and active targeting of overexpressed TFR (by Tf via receptor-mediated endocytosis). Moreover, the dual DOX-L exhibited higher cytotoxicity than all the other treatment groups in A2780 cancer cells and improved DOX accumulation in tumors. On the other hand, DOX has also accumulated at high levels in the liver and spleen with the treatment of dual DOX-L.

Another study that makes use of transferrin-targeted liposomes was that reported by Jhaveri and co-workers [67]. They developed Tf-targeted resveratrol-loaded liposomes for the treatment of glioblastoma, due to the limits of resveratrol (RES) physicochemical properties. The authors verified that the nanocarrier exhibited an enhanced in vitro activity, had a major ability to inhibit tumor growth and enhanced cell internalization.

**Figure 10:**
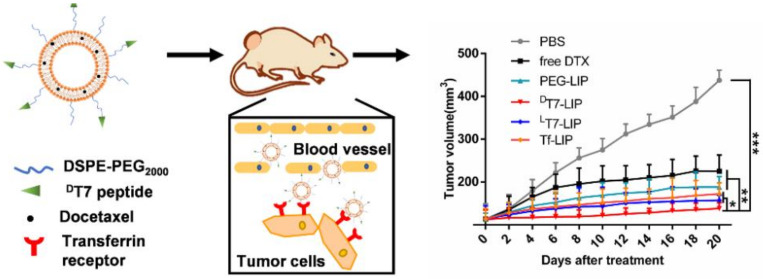
Comparison of efficacy, in vivo, of docetaxel (DTX)-loaded nanosystems compared with free-DTX in the treatment of hepatocellular carcinoma. Tumor growth until 20 days: phosphate buffer (PBS), effect of free docetaxel (free-DTX), effect of DTX-loaded nanosystems (^D^T7-LIP, ^L^T7-LIP, Tf-LIP and PEG-LIP); * *p* < 0.05, ** *p* < 0.01, *** *p* < 0.001 versus the ^D^T7-LIP group. Reprinted from [143] with permission from Elsevier, 2021.

Tang et al. [143] designed the new ^D^T7 peptide, a retro-inverso analog of ^L^T7 peptide, as a ligand for docetaxel-loaded liposomes, for targeted therapy of hepatocellular carcinoma. The study showed that the new targeted liposome (^D^T7-LIP) had better tumor accumulation than ^L^T7-LIP and Tf-LIP and exhibited a high binding affinity to TfR overexpressed on tumor cells. This DDS proved to be an alternative to improve the efficacy of docetaxel (DTX) in the treatment of hepatocellular carcinoma, since ^D^T7-LIP showed a significantly stronger in vitro and in vivo targeting (Figure 10). The authors hypothesized that ^D^T7 may be a potent ligand for designing DDS targeting TfR-overexpressing tumors.

- Targeting Folate Receptors with Liposomes

Folate receptors (FR) belong to the family of glycoproteins (35–40 kDa) and are highly overexpressed on the surface of many cancer cells [144,145]. Consequently, the folate receptor is a promising candidate for tumor-targeted delivery [144,146]. 

Liposomes functionalized with folate have been widely used to target FR in many cancers, including lung [147,148], ovarian [148,149], breast [39,150,151], colon [152] and lymphoma [148]. Patil et al. [148] developed folate targeted-PEGylated liposomes, loaded with a lipophilic prodrug of Mitomycin-C. Compared to non-targeted drug-loaded liposomes, the functionalized ones exhibited higher uptake by cancer cells with high folate receptors (HiFR). Accordingly, folate targeting resulted in increased cytotoxicity in vitro and more efficient growth inhibition of tumor cells. The authors verified that functionalized liposomes were taken up by cancer cells via folate receptor-mediated endocytosis.

**Figure 11:**
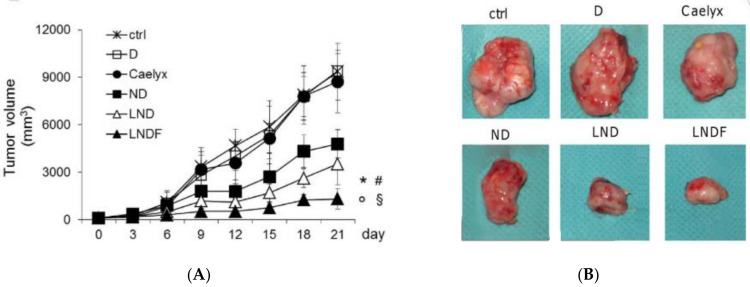
Enhanced anti-tumor effects of folate-targeted liposomal nitrooxy-DOXorubicin against resistant tumors. (**A**) Tumor growth until 21 days: control (ctrl); effect of free DOX (D); effect of Caelyx; effect of nitrooxy-DOXorubicin (ND); liposomal nitrooxy-DOXorubicin (LND); folate-targeted liposomal nitrooxy-DOXorubicin (LNDF). Data are presented as means ± SD. ND/LND/LNDF vs. ctrl group: * *p* < 0.01; ND/LND/LNDF vs. D: ° *p* < 0.01; LNDF vs. ND: # *p* < 0.001; LNDF vs. LND: § *p* < 0.05; (**B**) Pictures of representative tumors from each treatment group after mice sacrifice. Reprinted from [39] with permission from Elsevier, 2021.

Furthermore, Gazzano et al. [39] produced folate-targeted liposomes loaded with nitrooxy-DOXorubicin, decorated with folic acid (FA), intending to target P-glycoprotein (Pgp)-positive and FR-positive tumors. They verified that targeted liposomes reduced the growth of FR-positive/Pgp-positive tumors (Figure 11) and prevented tumor formation in mice and higher nuclear delivery. Moreover, targeted liposomes induced less cytotoxicity in MCF10A non-tumor cells than in MCF7 breast cancer cells, making them more selective for cancer cells. However, the non-targeted and targeted liposomes presented more accumulation in the liver than free DOX and nitrooxy-DOX. Still, such accumulation did not appear to be associated with liver damage. On the other hand, the liposomal formulations showed less accumulation than free drugs in the lungs.

- Targeting Lactoferrin Receptors

Lactoferrin (Lf) is a natural protein, 80 kDa glycoprotein, and belongs to the transferrin family [153,154]. Lactoferrin-based liposomes have been used as ligands of the lactoferrin receptor (LfR) to reach some cancers, such as liver and brain tumors [155,156], and as ligands of other overexpressed receptors [157,158]. Several recent studies demonstrated that Lf is a potential liposome ligand for targeting numerous receptors overexpressed in tumors, improving anticancer activity, increasing cellular uptake and simultaneously lowering side effects. Zhang et al. [158] proposed the use of Holo-lactoferrin (holo-Lf), a natural protein, as a potential ligand of the transferrin receptor. They developed a PEGylated liposome to load both holo-Lf and DOX. This system showed high cellular uptake and tumor accumulation, both in vitro and in vivo, in murine breast cancer (4T1 cell line) and great potential for cancer treatment using radiochemotherapy. Pireddu et al. [155] applied Lf as a ligand for liposomes to carry Triiodothyronine (T3), a thyroid hormone, and deliver it to hepatoma cells. In this case, the Lf-modified-liposomes specifically bind to LfR on the hepatoma cell’s surface, exhibiting sustained drug delivery and a reduced therapeutic dose, possibly avoiding the deleterious side effects associated with T3 treatment.

- Targeting of Tumoral Endothelium with Liposomes

Targeting overexpressed receptors in the endothelium of cancer cells is another approach of active targeting with nanoparticles, to prevent angiogenesis that contributes to cancer progression and metastasis [159].

- Vascular Cell Adhesion Molecule (VCAM)

Vascular cell adhesion molecule 1 (VCAM-1) belongs to the family of immunoglobulin (Ig) proteins and is overexpressed on the surface of endothelial cells in many cancers [160,161], and VCAM-1 can take responsibility for tumor proliferation and metastasis. Recently, VCAM-1 directed target-sensitive liposomes were developed by Calin et al. [162]. They developed PEGylated liposomes loaded with a CCR2 (C-C chemokine receptor 2) antagonist (Teijin compound 1), coupled to a specific peptide, with the amino acids sequence VHPKQHRGGSKGC (recognized by endothelial VCAM-1). The study revealed that the developed liposomes effectively release the entrapped drug after binding to activated endothelial cells [162].

- Integrins

Integrins are another class of receptors, belonging to the family of heterodimeric cell surface receptors, that contributes to cancer progression and tumor angiogenesis, via adhesion-dependent and -independent pathways. One particular integrin, α_V_β_3_, is overexpressed on many solid tumors and highly expressed on tumor vasculature, potentiating the activity of tyrosine kinase receptors. Further, it recognizes the tripeptide sequence RGD (Arg-Gly-Asp), present in the extracellular matrix proteins [163]. Bianchini et al. [164] developed liposomes functionalized with cyclo-aminoprolineRGD units to target integrin α_V_β_3_-overexpressing cells. The targeted liposomes, loaded with the anticancer drug sunitinib, were more efficient than the untargeted ones, both in in vivo and in vitro experiments. Moreover, the targeted liposomes showed greater uptake of sunitinib via receptor-mediated endocytosis and completely inhibited the formation of new blood vessels.

- Matrix Metalloproteinase

Matrix metalloproteinases (MMPs), zinc-dependent endopeptidases, are expressed in healthy fibroblasts, but also in almost all human cancer cells. These MMPs are capable of remodeling the extracellular matrix and promote angiogenesis, tumor growth, and metastasis, making them a viable target to treat cancer. Recent studies of drug delivery systems used MMPs as therapeutic targets in cancer [165].

Wei et al. [50] developed smart DOX-loaded liposomes carrying cilengitide, an α_V_β_3_ integrin-specific cyclic RGD-mimetic peptide, via a membrane-type 1 (MT1) matrix metalloproteinase cleavable peptide. The cleavage by MT1-MMP allowed improved accumulation, delivery, and therapeutic efficacy of DOX in pancreatic cancer. Involving the same inherent approach, Shi et al. [136] developed an intelligent “peptide-gathering mechanical arm” (Int PMA), with Asn-Gly-Arg (NGR) cell-penetrating peptide and PLGLAG peptide (a matrix metalloproteinase-2-cleavable peptide) to modify liposomes. The smart liposomes exhibited a sustained release in vitro and responded to endogenous and exogenous stimuli, enhancing the antitumor efficacy in vitro and in vivo.

Another study showed that PTX-loaded PEGylated cationic liposomes, modified with R8-dGR (RRRRRRRR-dGR) co-encapsulating pHIF-1α (plasmid hypoxia-inducible factor-1α), downregulated HIF-1α and its downstream molecules VEGF (vascular endothelial growth factor) and MMP-9, leading to enhanced antimetastatic effects in pancreatic cancer in vivo [166].

- Targeting Cell Organelles with Liposomes

After successful delivery into the targeted cell, a drug should get access to a particular organelle (endo/lysosome, mitochondria, Golgi apparatus, nucleus, etc.) to be effective. Organelle-specific delivery has become one of the primary goals for targeted drug delivery research [137]. Functionalized liposomes have considered intracellular targets, such as mitochondria, lysosomes and the nucleus.

- Mitochondrial Targeting

Mitochondria are fundamental for the metabolic functions of the cell due to their main function of producing energy, but these organelles also play a key role in mammalian cell death [167]. Many reports have been presented involving drug-loaded liposomes with mitochondria-targeting moieties, such as triphenylphosphonium (TPP) cation, dequalinium (DQA) [168], and mitochondria-targeting peptide sequence (MTS) [48,54].

Sun and co-workers [48] developed two targeted peptide-modified PEGylated lipids, based on the phospholipid DSPE, and including cyclic RGD (cRGD) or KLA (a peptide which leads to programmed cell death by disrupting the mitochondrial membrane). These two lipids were managed to make PTX-loaded liposomes. The study showed that the functionalized liposomes had a slightly negative surface charge, capable of reaching tumor tissue through the EPR effect. Moreover, the nanosystems provided high levels of cellular uptake (resulting in strong in vitro activity against 4T1 cells), anti-angiogenesis effects without systemic toxicity, and accumulation in mitochondria, allowing a mitochondria-mediated apoptosis pathway. 

Another investigation that used mitochondria-penetrating peptide (MPP) was reported by Mallick et al. [54]. They developed functionalized liposomes, loaded with Antimycin A (a hydrophobic drug), using the phenylalanine-arginine-phenylalanine-lysine (FRFK) peptide sequence to target mitochondria and lung cancer (A549 cells). Antimycin A showed a higher cytotoxic effect, leading to mitochondria-mediated apoptosis and enhanced bioavailability in cancer therapy. 

**Figure 12:**
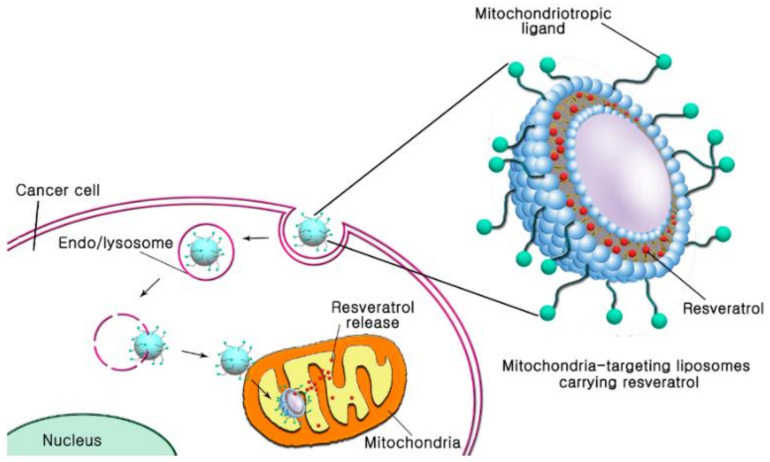
Schematic representation of mitochondria-targeting liposomes carrying resveratrol into mitochondria cancer cell. Reprinted from reference [168].

Another group of researchers [168] looked at a different targeting mitochondria approach, by using 4-carboxybutyl triphenylphosphonium bromide, or DQA attached to DSPE-PEG, as functional groups on the surface of liposomes (Figure 12). They verified that mitochondria-targeting liposomes, loaded with resveratrol, improved the drug cytotoxicity efficacy in B16F10 murine melanoma cells by ROS generation and mitochondrial depolarization, with increased accumulation in mitochondria and sustained delivery of resveratrol.

- Lysosomal Targeting

Lysosomes are membrane-bound intracellular organelles that receive macromolecules in cells, also containing different hydrolases. Lysosomes could be a therapeutic target, exploitable in the induction of apoptotic pathways in cancer cells [169]. Therefore, lysosomotropic ligand-targeted liposomes loaded with molecules capable to target cancer cells and destabilize lysosomes have been used to induce lysosomal membrane permeabilization and subsequent apoptosis. Minnelli et al. [170] used mannose-6-phosphate (M6P) moiety to target the mannose-6-phosphate/insulin-like growth factor receptor, overexpressed in many cancers and involved in the transport of cellular proteins from the cell surface or trans-Golgi network to lysosomes. They studied M6P-modified and non-functionalized liposomes in MCF7 tumor cells and HDF normal cells and verified that selective delivery of the active compound leads to an increased apoptotic effect in MCF7 cells. 

Hayward et al. [171] developed hyaluronic acid (HA) conjugated liposomes, which targeted human glioblastoma multiforme (GBM cells), with high expression of CD44 glycoproteins (HA receptor). The results demonstrated that this nanosystem allowed hyaluronic acid to promote preferential uptake, facilitate intracellular lysosomal evasion and enhance the chemotherapeutic potency in GBM cells.

- Nucleus Targeting

**Figure 13:**
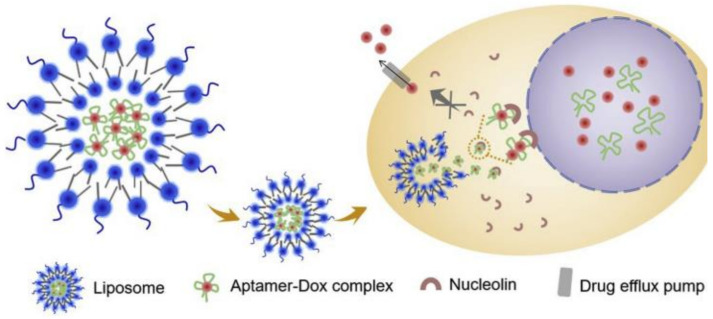
Release and accumulation of DOX in the cancer cell nucleus mediated by a modified liposome loaded with an aptamer-DOX complex. Reprinted from reference [172].

Modified nanocarriers can deliver drugs to the nucleus by targeting overexpressed nuclear proteins in cancer cells [172]. Nucleolin is a protein (overexpressed in many cancers) promoting angiogenesis and inhibiting apoptosis [173,174]. Aptamers are strong ligands used to target nucleolin protein and can enhance cellular uptake, via receptor-mediated endocytosis, enhance tumor accumulation and improve the pharmacokinetics of loaded drugs [174]. Therefore, Li et al. [172] developed a nuclear-targeted liposome loaded with and aptamer-DOX complex. The aqueous interior of liposome-containing DOX·HCl, inserted in the aptamer AS1411, revealed an enhanced accumulation and release of DOX in the nuclei of MCF-7/Adr cells (Figure 13). Also, the liposomal formulation allowed an enhanced antiproliferative activity when compared to free DOX. 

### 3.2. Functionalized Albumin Nanoparticles for Cancer Therapy

Albumin is a 66.5 kDa single-chain protein, containing 584–590 amino acid residues [175,176]. This water-soluble globular and natural biomacromolecule measures 6.67 ± 0.57 nm, being the most abundant protein in human blood plasma (~55%, 35–50 g/L), with an average half-life of ~19 days [176,177]. Human serum albumin possesses 35 cysteine residues, of which 34 give rise to disulfide bridges, contributing to its high stability [178].

Albumin is a biocompatible, biodegradable, non-toxic and non-immunogenic protein, possessing long blood circulation [177,179]. Tumor tissues present leaky vasculature and the absence of lymphatic drainage, which allows albumin to extravasate into the tumor tissue, being retained with high intratumor concentrations. Thus, albumin exhibits passive tumor targeting due to EPR effect. Moreover, two albumin-binding proteins—60 kDa glycoprotein (gp60) and albumin-binding protein SPARC (secreted protein acidic and rich in cysteine)—also enable cell uptake and retention of albumin by active targeting, because of their high affinity to the tumor interstitium [176,177,179]. Nevertheless, albumin binds metal ions Ni(II), Cu(II), Zn(II), and Ca(II) and acts as a great transport vehicle for these metal ions and other substances in the blood [119,180].

Due to all its unique and versatile properties, albumin nanoparticles have been used as nanocarriers of active biomolecules, such as drugs, peptides, proteins and nucleic acids. Drug nanocarriers have been produced using human serum albumin (HSA) or bovine serum albumin (BSA) [179,181]. Human serum albumin has risen as a great carrier for therapeutic agents, improving their pharmacokinetic profiles or delivering them to the tumor sites. Thus, a commercial PTX-loaded albumin formulation, Abraxane^®^, was approved by the U.S. FDA to treat some cancers [175].

**Figure 14:**
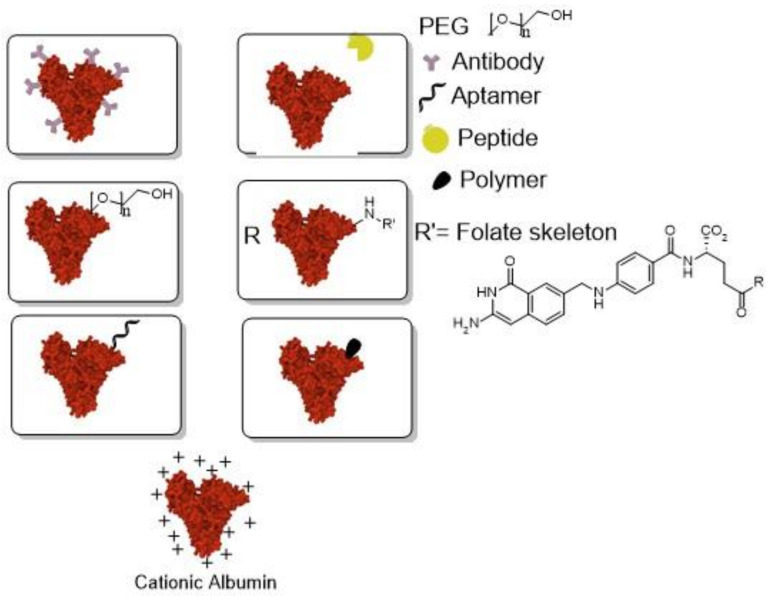
General representation of albumin with targeting ligands.

Albumin nanoparticles have many binding sites on the protein molecules; therefore, a drug can be conjugated with albumin via covalent bonding or just be adsorbed on its surface [181]. In addition to being able to shield therapeutic cargo until the delivery at the therapeutic site [182], albumin may be functionalized (Figure 14) with targeting ligands (antibodies, folate, peptides, aptamers), charged functional groups, or their combinations, for effective drug delivery, and target overexpressed receptors [183,184], as it will be explained hereafter. Recently, albumin-based nanocarriers have been reported as efficient DDS for cancer therapy [116,117,185]. These systems are also recognized for carrying hydrophobic drugs and enhancing their water solubility [118,130,186,187].

Albumin nanosystems have unique properties of targeting tumor cells, due to their possibility of improving in vivo drug distribution and reducing drug toxicity [32]. Thus, albumin nanocarriers can deliver drugs into tumor sites with the specific targeting of folate receptors, glycoproteins, growth factors, integrins and organelles.

Nevertheless, albumin based nanocarriers present some drawbacks, since their preparation implies the use of toxic cross-linking reagents to increase their stability to prevent a burst release of drugs; their efficacy as drug carriers may be defined by the net negative charge of albumin under physiological conditions. Yet, albumin can be modified with different functional groups, such as carboxyl, hydroxyl or amino groups, and improve the efficiency of the delivery system [118,130,188,189].

#### 3.2.1. Targeting Overexpressed Receptors on Cancer Cells with Albumin

- Targeting Growth Factors with Albumin

Functionalized albumin nanocarriers can target overexpressed growth factors, such as HER2 and EGFR. Wan et al. [190] encapsulated lapatinib, a dual-tyrosine kinase inhibitor of human epidermal growth factor receptor (HER2) and EGFR, because of its low oral bioavailability. Thus, the authors developed lapatinib-loaded HSA nanoparticles without any modification of albumin. The nanoparticles effectively inhibited the adhesion, migration and invasion ability of brain-metastatic 4T1 cells. 

However, other ways to target overexpressed growth factors with albumin nanoparticles have been used. In the study of Saleh et al. [118], HB5 aptamer, with high selectivity and binding affinity to HER2, was used to develop an aptamer-decorated curcumin-loaded HSA nanoparticle, with the capacity to target HER2-positive breast cancer cells and to increase the water solubility of the drug by 400-fold. Further, they verified a remarkable cytoplasmic uptake and high cytotoxicity of these albumin nanoparticles in HER2-overexpressing SK-BR-3 cells.

Santos-Rebelo et al. [191] developed parvifloron D (PvD)-loaded albumin nanoparticles. To target EGFR-overexpressing pancreatic cancer cells, they attached the drug erlotinib and the antibody cetuximab to BSA nanoparticles’ surface and evaluated the antiproliferative effects in BxPC3 and Panc-1 cell lines. They verified that erlotinib-conjugated PvD-loaded BSA NPs presented the highest antiproliferative effect toward pancreatic tumor cells.

- Targeting Folate Receptor with Albumin

**Figure 15:**
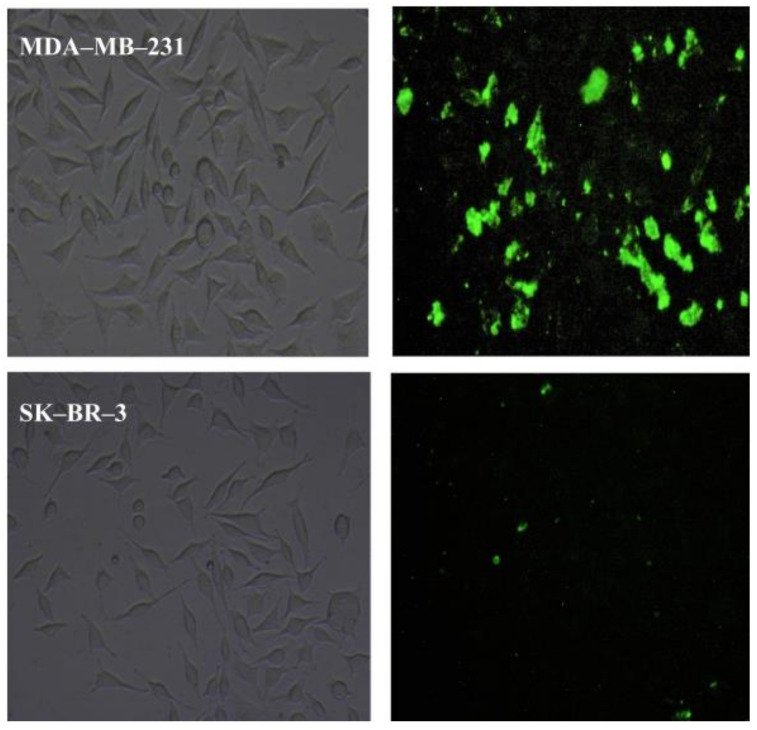
Fluorescence microscopy images of folate-conjugated ARM-HSA NPs in treated MDA-MB-231 cells (high FRα-expressing cell line) and SK-BR-3 cells (low FRα-expressing cell line). Reprinted from [130] with permission from Elsevier, 2021.

Akbarian et al. [130], to target folate receptor α (FRα), developed folate-decorated HSA nanoparticles to deliver artemether (ARM), which is characterized by its poor solubility and bioavailability. Firstly, the drug encapsulated in HSA NPs allowed an improvement of its water solubility by 50-fold. Then, the ligand folate was incorporated in ARM-HSA NPs, which allowed enhanced targeted delivery to FRα-overexpressing MDA-MB-231 breast cancer cells, due to FRα-mediated endocytosis (Figure 15).

Dubey et al. [189] developed an albumin-based nanocarrier for gemcitabine, because of its short half-life and side effects. The gemcitabine-loaded folate-functionalized BSA nanoparticles showed enhanced anticancer activity against folate receptor overexpressing cell lines (Ovcar-5 and MCF-7) compared to non-targeted nanoparticles, while the cytotoxicity in the folate receptor-deficient cell line (MIAPaCa-2 cells) was meaningless. In addition, the folate-functionalized nanoparticles enhanced cellular uptake of gemcitabine into the cells via folate receptors, and albumin-based nanocarriers protected the drug from in vivo degradation.

Meng et al. [32] also developed folate (FA)-decorated albumin nanoparticles. They attached FA-PEG to albumin as a targeting ligand, and the functionalized albumin showed increased delivery efficiency of cabazitaxel (CTX) and enhanced cytotoxicity against HeLa cells. The results indicated that the mediated cellular uptake of CTX-loaded FA-PEG functionalized albumin NPs could be enhanced by folate receptors. Other studies using folate-based albumin nanoparticles showed an enhanced folate receptor-mediated targeted uptake or prolonged blood circulation time in various types of cells, such as liver, breast, lung, ovarian and cervical cancer cells [186,192,193].

#### 3.2.2. Targeting Glycoproteins with Albumin

Another way to target cancer cells is targeting glycoproteins, such as SPARC and CD44 [40,194]. SPARC or osteonectin is an albumin-binding glycoprotein that mediates interactions between cells and their extracellular surrounding during morphogenesis, tissue remodeling and angiogenesis [195]. 

Zhao et al. [40] developed mannosylated albumin NPs with co-encapsulated disulfiram/copper complex and regorafenib. The study indicated that the modified albumin NPs targeted the drug-resistant colon cancer cells (HCT8/ADR) and their microenvironment via multiple nutrient transporters, such as SPARC and mannose receptors, and displayed higher uptake efficiency than the non-modified albumin NPs. However, modified albumin NPs displayed considerable accumulation in the liver, but less than co-encapsulated disulfiram/copper complex and regorafenib, which indicated a reduction in side effects using this nanocarrier.

CD44, a family of non-kinase single transmembrane glycoproteins, involved in several intracellular pathways, are highly expressed in gastrointestinal, prostate, breast and pancreatic cancer cells. Hyaluronic acid, peptides and aptamers have been used as ligands in nanocarriers to deliver chemotherapeutic agents, maximizing the drug effect on CD44 overexpressing cells [196]. Hyaluronic acid was recently used [187,194] as a ligand of albumin. Edelman and collaborators [187] entrapped PTX and imidazoacridinones, both hydrophobic drugs, into BSA nanoparticles decorated with HA. These targeted albumin NPs were more cytotoxic to ovarian cancer cells overexpressing CD44 (SKOV3 cells) than free PTX, but not to cells with low expression of CD44, which provides evidence for a CD44-mediated uptake of the PTX-loaded NPs. Another study [194] also demonstrated that HA on the surface of albumin nanoparticles allowed high affinity and specific binding to CD44-enriched melanoma B16F10 cells. 

#### 3.2.3. Targeting Integrins with Albumin

**Figure 16:**
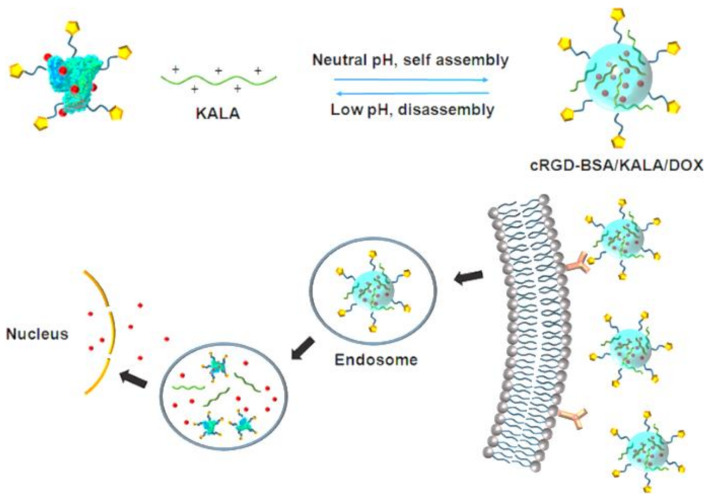
Illustration of the formation of cRGD-BSA/KALA/DOX nanoparticles and of the efficient delivery of DOX, including cellular uptake mediated by cRGD, cell internalization and pH-triggered disassembly to accelerate DOX release. Reprinted from [197] with permission from American Chemical Society, 2021.

To target the overexpressed integrin α_V_β_3_, Chen et al. [197] developed a dual peptide-functionalized BSA-based nanocarrier, using cRGD and the cationic cell-penetrating peptide KALA, to deliver doxorubicin hydrochloride and evaluated the tumor-targeting delivery using U87-MG glioblastoma cells. The nanocarrier was developed based on the self-assembly between the cationic KALA and BSA functionalized with cRGD as the tumor-targeting ligand. The functionalized nanocarrier enhanced cell internalization due to the integrin targeting of cRGD and the cell-penetrating effect of KALA (Figure 16). The pH-triggered changes in the electric charges of cRGD-BSA and KALA led to the disassembly of the nanoparticles to accelerate intracellular drug release. Thus, the targeted nanocarriers have enhanced cytotoxicity against U87-MG cells compared to free DOX, due to increased growth inhibition of tumor cells with overexpressed α_V_β_3_-integrin.

Another group of researchers [198] also reported the use of RGD modification and developed HSA nanoparticles loaded with PTX, using Chlorin e6 (Ce6), a photosensitizing agent, and RGD peptide. Two types of nanoparticles were produced, one using co-assembly of HSA-Ce6 and HSA-RGD simultaneously and another with a HSA-Ce6@HSA-RGD core-shell structure. Both types of NPs can target α_v_β_3_-integrin positive tumor cells (U87MG cells) in vitro. Moreover, synergistic cancer killing was observed when the cells treated with these NPs were exposed to light irradiation, by the photodynamic-effect-induced endosomal drug release. However, the produced nanoparticles showed some significant accumulation in the liver, kidney, and lung.

#### 3.2.4. Targeting Organelles in Tumor Cells with Albumin

Functionalized albumin nanoparticles can target tumor organelles (nucleus or mitochondria) the same way as liposomes. Xu et al. [199], aiming to target nucleolin, used the hydrophobic drug DOX to trigger the self-assembly of BSA, forming stable nanoparticles via hydrophobic interaction. Then, a nucleolin-targeting aptamer (AS1411) was incorporated in the NPs’ surface. The study revealed that aptamer-modified BSA NPs presented higher cellular uptake and inhibitory effect against MCF-7 cancer cells compared to non-targeted nanoparticles, due to the specific recognition of AS1411 by nucleolin overexpressed on tumor cells. Besides, the aptamer had a unique property of effectively inducing cell apoptosis, by down-regulation of the Bcl-2 apoptosis regulator and PCNA (proliferating cell nuclear antigen) in MCF-7 cells. Another group of researchers [200] also explored albumin nanoparticles functionalized with AS1411, but carrying DTX, which presented a sustained release profile. The results showed that functionalized nanoparticles were preferentially ingested by nucleolin-expressing CT26 colon cancer cells, with an enhanced cell killing effect. The targeted NPs improved antitumor efficacy without raising systemic toxicity when compared to non-targeted NPs.

On the other hand, Battogtokh et al. [201] conjugated DTX with 4-carboxybutyl triphenylphosphonium (TPP) and developed TPP-DTX-loaded folate-cholesteryl-BSA NPs. They studied mitochondrial accumulation, in vitro cytotoxicity and in vivo antitumor activity, verifying that TPP-DTX selectively accumulated in mitochondria after internalization in the cells. The developed nanosystem showed enhanced tumor growth inhibition, both in vitro (MCF7 breast cancer cells) and in vivo, compared to the free drug. The authors confirmed that dual targeting (mitochondrial and folate receptor) could increase the therapeutic efficiency of anticancer drugs.

## 4. Conclusions

Conventional cancer therapy has several problems, such as strong side effects, low solubility of drugs, and multi-drug resistance. To overcome these problems, nanosystems have been used to increase the solubility and efficacy of drugs. When target strategies are applied, passive and active approaches have been used. The active targeting has been the most used recently, by applying different ligands in the nanocarriers’ surface to target specific receptors, glycoproteins or even organelles, present in cancer cells.

A large number of studies have proved that functionalized liposomes and albumin-based nanocarriers can successfully deliver hydrophobic (or hydrophilic) drugs into cancer cells through different targeting approaches. In summary, functionalized nanosystems have been revealed to be fundamental to improve antitumor activity and drug uptake, to avoid premature drug release, to decrease systemic toxicity and overcome multi-drug resistance.

## Data Availability

Not applicable.
